# In Overweight or Obese Pregnant Women, Maternal Dietary Factors are not Associated with Fetal Growth and Adiposity

**DOI:** 10.3390/nu10070870

**Published:** 2018-07-05

**Authors:** Cecelia M. O’Brien, Jennie Louise, Andrea Deussen, Jodie M. Dodd

**Affiliations:** 1 School of Paediatrics and Reproductive Health, and Robinson Research Institute, University of Adelaide, Adelaide 5006, Australia; jennie.louise@adelaide.edu.au (J.L.); andrea.deussen@adelaide.edu.au (A.D.); jodie.dodd@adelaide.edu.au (J.M.D.); 2 School of Public Health, University of Adelaide, Adelaide 5006, Australia; 3 Department of Perinatal Medicine, Women’s and Babies Division, Women’s and Children’s Hospital, Adelaide 5006, Australia

**Keywords:** obesity, pregnancy, fetal biometry, adiposity, healthy eating index, total energy, glycaemic index, protein intake, carbohydrate intake, fat intake

## Abstract

The aim of our study was to evaluate associations between maternal dietary factors and fetal growth and adiposity in overweight and obese women. Women randomised to the ‘Standard Care’ group of the LIMIT trial were included. Maternal dietary factors including Healthy Eating Index, total energy, fat, carbohydrates, protein, glycaemic load and index were measured using the Harvard semi-quantitative Food Frequency questionnaire at time of study entry, 28 and 36 weeks’ gestation. Fetal ultrasound measurements of biometry and adiposity were obtained at 28 and 36 weeks’ gestation. Linear regression models were used to associate between dietary factors and fetal growth and adiposity measurements. There were 721 women included in this exploratory analysis. A 10 unit increase in the log total energy was associated with a reduction in mid-thigh lean mass by 4.94 mm at 28 weeks (95% CI −9.57 mm, −0.32 mm; *p* = 0.036) and 7.02 mm at 36 weeks (95% CI −13.69 mm, −0.35 mm; *p* = 0.039). A 10 unit increase in Healthy Eating Index score was associated with a reduced mean subscapular skin fold measure at 28 weeks by 0.17 mm (95% CI −0.32 mm, −0.03 mm; *p* = 0.021). We did not identify consistent associations between maternal diet and measures of fetal growth and adiposity in overweight and obese women.

## 1. Introduction

Over the past 40 years, rates of obesity have tripled worldwide [1], to the extent that it is considered a public health crisis [2]. In many developed countries, including the United Kingdom and United States of America, 1 in 2 women now enter pregnancy overweight or obese [3,4,5]. There are well-recognised independent associations between obesity in pregnancy and maternal, fetal and neonatal health outcomes [6,7], and in the longer-term maternal obesity has been linked with childhood obesity [8].

Women who are overweight or obese during pregnancy have been demonstrated to have poorer diet quality when compared with women with BMI in the normal range [9,10,11,12], which persists into the postpartum period [10]. In turn, poor diet quality is associated with increased risk of glucose intolerance and pre-eclampsia [11], increased neonatal adiposity [13] and changes in child body composition [14].

There is growing interest in the programming of fetal growth, the critical time points and the influence of maternal diet as a potentially modifiable factor. The current literature is inconsistent, largely due to the heterogeneity and variability relating to the timing and types of dietary assessments, reporting and methodology along with body composition outcome measurements [15]. In relation to maternal carbohydrate intake for example, some studies have shown a positive effective of high carbohydrates on childhood BMI [16] and others have shown a low carbohydrate diet was associated with increased fetal abdominal fat [17]. The majority have shown a negative effect [18,19,20] and one study has shown no effect [15]. Protein and carbohydrate ratios or combination diets have also been reported, where high protein associated with low carbohydrate and fat diet was associated with a reduction in neonatal abdominal adiposity [21], whereas another study showed low protein: carbohydrate ratio was associated with increased abdominal fat in the fetus.

Several studies have explored the association between maternal dietary intake and outcomes in the perinatal period, focussing predominantly on birthweight [22,23,24,25], preterm birth [26], infants born small for gestational age [27], and newborn anthropometry [13,17,18,22,28,29]. Poor diet quality (defined as Healthy Eating Index score less than or equal to 57) has been associated with a higher percentage of neonatal fat mass as measured on air displacement plethysmography, independent of maternal BMI [13]. Observational data from the Danish National Birth Cohort identified an association between maternal dietary glycaemic load and both an increased risk of large for gestational age infants (14%) and higher birthweight (36 grams) [29].

There has been more limited evaluation of the contribution of maternal dietary intake to fetal growth and adiposity. Maternal protein, fatty acid and carbohydrate intake during pregnancy have all been associated with increased measures of fetal adiposity, although this has been evaluated in women of normal BMI [17]. The contribution of specific maternal dietary components to fetal growth and adiposity among women who are overweight or obese is unclear, and warrants further investigation. 

The aim of our study was to evaluate associations between maternal dietary factors and fetal growth and adiposity measured by ultrasound at 28 and 36 weeks gestation in overweight and obese women.

## 2. Materials and Methods 

The study cohort involves 721 overweight or obese pregnant women who participated in the Standard Care Group of the LIMIT trial. The methodology and findings of the LIMIT Trial have been reported in detail previously [30,31]. Briefly, women were recruited from maternity hospitals in South Australia, after ethics approval and informed written consent to participate. Eligible women were those with a singleton pregnancy and BMI ≥ 25 kg/m^2^ at booking antenatal appointment, and who were between 10^+0^ and 20^+0^ weeks’ gestation. The exclusion criteria included women with Type 1 or 2 Diabetes diagnosed prior to pregnancy or multiple pregnancy. At the booking antenatal appointment, all women had their height and weight measured, and BMI calculated by clinical staff. Women in the Standard Care group continued pregnancy care according to the guidelines of their local hospital and did not include specific information relating to weight gain, or diet and physical activity during pregnancy. The ethics approval study number for LIMIT randomised controlled trial was 1839/6 (approved July 2006) and for the fetal growth ancillary study the number was 2051/4 (approved April 2008).

### 2.1. Dietary Assessment

Women completed the Harvard Semi-quantitative Food Frequency (Willett) questionnaire [32] to measure the daily dietary intake of nutrients from 126 food items, including portion size and incorporation of the 7 food groups, which has been validated in pregnancy [33] and among Australian pregnant women [34]. The questionnaire was completed at the time of study entry, 28 and 36 weeks’ gestation. At study entry, women were asked on average, how often was the food consumed during the last 12 months, while assessment at 28 and 36 weeks’ gestation asked women to indicate on average, how often the amount of food was consumed since the previous questionnaire time point.

Daily nutrient intake was estimated using the nutrient compositions from the Australian food composition tables according to pre-specified portion size. Adherence to dietary recommendations was performed by allocating all food and drink consumption into the food groups as described by the Australian Guide to Healthy Eating [35]. Foods were classified as ‘non-core foods’ if the food did not meet the criteria of the five core food groups, provided minimal nutrient content, and were high in fat, sugar or salt [35,36].

Micronutrient values were obtained from the Harvard Semi-quantitative Food Frequency (Willett) questionnaire [32] and analysed as mean intake, utilising the Food Works Nutrient Analysis Software Package (FoodWorks, version 7, Professional; Xyris Software 2012; Australia), and using Australian Food composition tables.

Diet quality was assessed using the Healthy Eating Index (HEI), which has 12 components to yield a maximum score of 100 [37]. These 12 components include total fruit, total vegetables, dark green and orange vegetables and legumes, total grains and whole grains, all of which receive a score out of 5. Milk, meat and beans, oils, saturated fat and sodium-based foods were scored out of 10. Calories from solid fats, alcohol related beverages and added sugars were scored out of 20. A HEI score of 80 is considered good, a score between 50 and 80 is one that needs improvement, and scores of less than 50 are considered poor. The HEI has been validated for use in pregnant women [38]. 

Dietary glycaemic index (GI) values were obtained from the Harvard Semi-quantitative Food Frequency (Willett) questionnaire [32] and analysed using the Food Works Nutrient Analysis Software Package (FoodWorks, version 7, Professional; Xyris Software 2012; Brisbane, Australia), and published dietary glycaemic index values.

### 2.2. Ultrasound Assessment

A research ultrasound scan was offered to all women participating in the study at approximately 28 and 36 weeks’ gestation. Fetal biometry and body composition measurements were obtained as previously described [39]. Research ultrasounds were performed by medical practitioners with specialist or subspecialist training in obstetric ultrasound, while blinded to the participant’s treatment allocation, and all measurements were obtained prospectively. The estimated date of confinement and gestational age were calculated on the early pregnancy clinical ultrasound and menstrual period dating.

Ultrasound measurements of biometry and fetal adiposity were obtained as described in detail previously [39]. In brief, fetal biometry was measured at 28 and 36 weeks’ gestation. This included head circumference (HC), biparietal diameter (BPD), abdominal circumference (AC) and femur length (FL), measured in accordance with national and international standards of practice [40]. Estimated fetal weight (EFW) was calculated using the Hadlock C formula [41]. 

Fetal body composition measures included mid-thigh lean mass (MTLM), mid-thigh fat mass (MTFM), abdominal fat mass (AFM), and subscapular fat mass (SSFM) using techniques reported previously [39]. The techniques for acquisition of these measurements have been published in detail [39]. MTLM was calculated by tracing the circumference of the mid-thigh total mass (MTTM) followed by the MTLM incorporating muscle and bone. A subtraction was performed between the MTTM and the MTLM to calculate the mid-thigh fat mass (MTFM). Abdominal fat mass was measured in millimetres between the mid-axillary lines and anterior to the margins of the ribs, at the level of the abdominal circumference. Two measurements of the subcutaneous skin width were obtained from a longitudinal section of the scapula at the interface with the super-spinous and infra-spinous muscles.

### 2.3. Statistical Analysis

Baseline characteristics of women contributing data were assessed descriptively. Continuous variables were reported as mean and standard deviation or median and interquartile range as appropriate, and categorical variables as a number and percentage. For each fetal biometry measurement, *z* scores were calculated using ultrasound growth charts in clinical use [41,42].

The analyses were exploratory, with no pre-specification of a primary outcome. Instead, the associations between diet and fetal growth and adiposity were investigated using a range of dietary variables (HEI, Total Energy, Glycaemic Index, Glycaemic Load, Fat, Carbohydrate and Protein as Percent of Total Energy) and a range of fetal growth and adiposity measures (BPD and BPD *z*-score, EFW and EFW *z*-score, HC and HC *z*-score, AC and AC *z*-score, FL and FL *z*-score, MTLM, MTFM, AFM, SSFM). Both dietary and fetal growth variables were measured at 28 weeks’ and 36 weeks’ gestation.

Linear regression was used to model the association between dietary factors and fetal growth and adiposity, with diet variables considered as ‘predictors’ (independent variables) and fetal growth and adiposity variables as ‘outcomes’ (dependent variables). A time-by-diet-variable interaction term was included to allow for estimation of the association at each time point separately, and to test whether the association differed between time points. Generalised Estimating Equations with exchangeable working correlation were used to account for repeated measures. Both unadjusted and adjusted analyses were performed. Adjusted analyses included maternal BMI category (25.0–29.9 kg/m^2^ vs. ≥30.0 kg/m^2^ as measured at study entry), smoking, parity (0 vs. ≥1), age and Socio-Economic Indexes for Areas Index of Relative Socio-Economic Disadvantage (SEIFA IRSD) quintile, which is a rank of areas within Australia according to socio-economic disadvantage, obtained from the Census that occurs every 5 years. All analyses were additionally adjusted for baseline diet variables, as a potential confounder.

Statistical significance was assessed at the two-sided *p* < 0.05 and no adjustment was made for multiple comparisons. All analyses were performed using SAS 9.4 (Cary, NC, USA). 

## 3. Results

### 3.1. Demographic Characteristics

There were 721 women included in this secondary analysis and the baseline characteristics are shown in Table 1. The mean age of women participating was 29.9 years (SD 5.3), with median gestation at study entry 14.3 weeks (Interquartile range between 12.1 to 17.0 weeks). Forty-three percent (*n* = 310) of women were overweight, while 46.5% (*n* = 335) were obese (BMI 30.0–39.9 kg/m^2^), and 10.5% (*n* = 76) morbidly obese (BMI ≥ 40.0 kg/m^2^). Most women (91%; *n* = 659) were of Caucasian origin, 41.3% (*n* = 298) in their first ongoing pregnancy, and 52% (*n* = 373) from the highest two quintiles of social disadvantage. The baseline characteristics of the women contributing dietary and ultrasound data were comparable to all women in the Standard Care group, and all women included in the LIMIT randomised trial [30].

### 3.2. Healthy Eating Index (HEI)

There were no consistent associations between HEI and fetal biometry, MTFM, MTLM and AFM (Table 2). There was a negative association between HEI and SSFM at 28 weeks, whereby a 10-unit increase in HEI reduced SSFM by 0.17 mm (95% CI −0.32 to −0.03; *p* = 0.021).

### 3.3. Log Total Energy

Total Energy was log-transformed for analysis due to substantial right skew. There were no associations between log total energy and AC, EFW, all fetal biometry *z*-scores, MTFM, AFM and SSFM (Table 3). There was a negative association with log total energy and biometry measurements of BPD and HC at 36 weeks, such that a 10 unit increase in log total energy reduced BPD by 1.48 mm (95% CI −2.55 mm to −0.40 mm; *p* = 0.007); and HC by 4.07 mm (95% CI −7.6 mm to −0.54 mm; *p* = 0.024). 

At 28 and 36 weeks’ gestation, there were negative associations between log total energy and MTLM, such that a 10-unit increase in log total energy reduced MTLM by 4.94 mm (95% CI −9.57 mm to −0.32 mm; *p* = 0.036) at 28 weeks; and by 7.02 mm (95% CI −13.69 mm to −0.35 mm; *p* = 0.039) at 36 weeks. 

### 3.4. Glycaemic Index

There were no associations between dietary Glycaemic Index and fetal biometry including HC, FL, AC and EFW, related *z* scores and adiposity measures (Table 4). A negative association was identified between dietary glycaemic index and fetal BPD and its *z*-score, such that a 10-unit increase in dietary glycaemic index reduced BPD by 0.11 mm (95% CI −0.21 mm to −0.01 mm; *p* = 0.035), and BPD *z*-score by 0.35SD (95% CI −0.69SD to −0.01SD; *p* = 0.045) at 28 weeks.

### 3.5. Glycaemic Load

There were no consistent associations between dietary glycaemic load and fetal biometry, *z*-scores or adiposity measures at either 28 or 36 weeks (Table 5).

### 3.6. Fat, Carbohydrate and Protein as a Percent of Total Energy

There were no associations identified between fat as shown in Table 6.

There were no consistent associations between carbohydrate (Table 7) and protein intake (Table 8) and fetal biometry, *z*-scores or adiposity measures at either 28 or 36 weeks.

## 4. Discussion

The objective of this secondary exploratory analysis [30], was to determine if maternal dietary factors were associated with fetal body composition in women entering pregnancy overweight or obese. Our analysis found an increase in total energy of the maternal diet was associated with a reduction in mid-thigh lean mass of the fetus. Secondly, an increase in the Healthy Eating Index was associated with a reduction in the subscapular fat mass. While these individual associations were statistically significant, the actual differences were of small magnitude and were unlikely to be of clinical significance. Overall, we did not identify consistent associations between maternal diet and fetal growth or adiposity.

To our knowledge, this is the first study to describe the relationship between maternal dietary factors and fetal body composition in women entering pregnancy overweight and obese. There has been one study to describe the maternal dietary factors and fetal adiposity measurements in 179 women with a normal BMI [17]. This study measured different dietary variables including a derived ratio comparing protein and carbohydrate, and poly-unsaturated fatty acids as a percentage of energy intake. The authors also described a variation in ultrasound techniques for the measurement of fetal adiposity [17]. Women with lower dietary protein intake demonstrated higher abdominal wall adiposity, while fetal thigh adiposity was greatest among women whose diet consisted of low carbohydrate, intermediate protein and high fat intake [17].

The majority of the literature relates to neonatal and infant body composition [15,21,24], birthweight [18,19,20] with variable methodology and inconsistent findings [21,43]. An explanation for the lack of association seen in our study and inconsistent findings within the literature may relate to the timing of the dietary assessment. Early 2nd trimester maternal dietary analysis has been assessed in the literature [15,44] with no consistent findings [15,20,24,44]. One study assessed dietary intake between 8 and 12 weeks and found carbohydrate consumption was associated with increase in birthweight, whereas fat intake was associated with lower birthweight [19]. It is also likely that the fetal programming of infant growth patterns is much more complex, with the impact of epigenetics, paternal factors, postnatal environment [45].

The main strength of our secondary analysis relates to the large sample size of women entering pregnancy overweight or obese. The data was derived from the largest randomised controlled trial utilising robust methodology [30] and the first to measure the effect of an antenatal intervention on fetal biometry and adiposity [39]. The main limitation of the current analysis is the reliance on self-reported measurements of maternal dietary intake. Dietary analysis is subject to multiple biases including measurement error, recall bias related to the food questionnaire, along with reporting bias. A comparator group of women entering pregnancy with a normal BMI would have also added valuable data, including a baseline for assessment of both fetal growth patterns and maternal dietary intake.

Several randomised trials have identified improvements in maternal dietary patterns during pregnancy following provision of a lifestyle intervention [30,31,46,47,48,49]. The LIMIT trial demonstrated that the provision of the antenatal lifestyle and dietary intervention improved women’s intake of fibre, saturated fat, fruits and vegetables and micronutrient intake, although did not impact overall energy intake [31]. Other trials have also shown significant improvements in maternal diet, physical activity [31,46,47,48] and insulin resistance [46,50].

While individual trials conducted in overweight and obese pregnant women have described positive effects on maternal dietary and lifestyle behaviours [51], intervention trials overall have generated disappointing results in terms of clinical pregnancy and birth outcomes. Whether relatively modest improvements in maternal diet are sufficient to impact fetal adiposity measures, which themselves are relatively insensitive indices, remains to be determined [52,53]. Furthermore, there is evidence to suggest that fetal growth and adiposity may be programmed much earlier in gestation than current interventions have targeted [54], highlighting the importance of optimal diet and maternal weight prior to conception [2,55,56,57].

There is growing interest in strategies to optimise both maternal and paternal dietary intake and weight in the peri-conceptual period [3,58,59]. This primary prevention strategy may reduce the intergenerational transmission of obesity from mother to child and may improve pregnancy outcomes [2,45,60]. Further studies are required to understand the timing of and factors relating to programming of fetal growth and body composition.

## Figures and Tables

**Table 1:** Baseline characteristics of the Standard Care group within the LIMIT Trial.

Characteristic	Number (%), Mean (SD) or Median (IQR)
Overall Number	721
- Both 28 and 36 Weeks	453
- 28 Weeks Only	158
- 36 Weeks Only	110
Age at Trial Entry: Mean (SD)	29.88 (5.33)
Parity: *N* (%)	
- 0	298 (41.33)
- ≥1	423 (58.67)
BMI: Median (IQR)	31.00 (27.70, 35.20)
BMI Category: *N* (%)	
- BMI 25.0–29.9	310 (43.00)
- BMI 30.0–34.9	219 (30.37)
- BMI 35.0–39.9	116 (16.09)
- BMI ≥ 40.0	76 (10.54)
Smoker: *N* (%)	
- Yes	67 (9.29)
- No	639 (88.63)
- Unknown	15 (2.08)
GA at Trial Entry: Median (IQR)	14.29 (12.14, 17.00)
Public Patient: *N* (%)	
- Yes	707 (98.06)
- No	14 (1.94)
Ethnicity: *N* (%)	
- Caucasian	659 (91.40)
- Asian	22 (3.05)
- Aboriginal or TSI	8 (1.11)
- Indian, Pakistani, Sri-Lankan	22 (3.05)
- African	5 (0.69)
- Other	5 (0.69)
SEIFA IRSD Quintile: *N* (%)	
- Quintile 1	199 (27.60)
- Quintile 2	174 (24.13)
- Quintile 3	117 (16.23)
- Quintile 4	116 (16.09)
- Quintile 5	115 (15.95)

**Table 2:** Healthy Eating Index and fetal ultrasound measurements.

Outcome	Unadjusted Estimate (95% CI)	Unadjusted *p* Value	Adjusted Estimate (95% CI)	Adjusted *p* Value
BPD		0.992 ^†^		0.968 ^†^
- 28 Weeks	−0.03 (−0.08, 0.02)	0.239	−0.03 (−0.08, 0.02)	0.221
- 36 Weeks	−0.03 (−0.08, 0.02)	0.235	−0.03 (−0.08, 0.02)	0.234
BPD *z*-score		0.728 ^†^		0.645 ^†^
- 28 Weeks	−0.13 (−0.29, 0.04)	0.128	−0.13 (−0.30, 0.03)	0.117
- 36 Weeks	−0.10 (−0.24, 0.04)	0.166	−0.09 (−0.24, 0.05)	0.194
HC		0.060 ^†^		0.064 ^†^
- 28 Weeks	−0.06 (−0.22, 0.09)	0.425	−0.08 (−0.24, 0.08)	0.305
- 36 Weeks	0.10 (−0.05, 0.09)	0.194	0.08 (−0.08, 0.24)	0.313
HC *z*-score		0.026 ^†^		0.025 ^†^
- 28 Weeks	−0.06 (−0.17, 0.05)	0.317	−0.07 (−0.18, 0.04)	0.210
- 36 Weeks	0.08 (−0.03, 0.05)	0.161	0.07 (−0.05, 0.18)	0.247
FL		0.168 ^†^		0.211 ^†^
- 28 Weeks	−0.02 (−0.06, 0.02)	0.283	−0.02 (−0.06, 0.02)	0.232
- 36 Weeks	0.01 (−0.03, 0.02)	0.646	0.00 (−0.03, 0.04)	0.855
FL *z*-score		0.097 ^†^		0.116 ^†^
- 28 Weeks	−0.09 (−0.20, 0.03)	0.154	−0.09 (−0.21, 0.03)	0.131
- 36 Weeks	0.02 (−0.09, 0.03)	0.678	0.01 (−0.10, 0.13)	0.817
AC		0.927 ^†^		0.976 ^†^
- 28 Weeks	−0.07 (−0.28, 0.13)	0.484	−0.13 (−0.33, 0.07)	0.210
- 36 Weeks	−0.06 (−0.32, 0.13)	0.637	−0.13 (−0.38, 0.13)	0.338
AC *z*-score		0.712 ^†^		0.691 ^†^
- 28 Weeks	−0.03 (−0.14, 0.09)	0.660	−0.06 (−0.18, 0.05)	0.264
- 36 Weeks	−0.05 (−0.18, 0.09)	0.471	−0.09 (−0.22, 0.04)	0.193
EFW		0.512 ^†^		0.562 ^†^
- 28 Weeks	−22.42 (−52.33, 7.48)	0.142	−30.24 (−60.55, 0.07)	0.051
- 36 Weeks	−6.80 (−58.91, 7.48)	0.798	−16.24 (−68.74, 36.27)	0.544
EFW *z*-score		0.344 ^†^		0.366 ^†^
- 28 Weeks	−0.08 (−0.18, 0.03)	0.170	−0.10 (−0.21, 0.01)	0.063
- 36 Weeks	−0.02 (−0.14, 0.03)	0.723	−0.05 (−0.17, 0.07)	0.415
MTLM		0.742 ^†^		0.891 ^†^
- 28 Weeks	−0.07 (−0.25, 0.10)	0.417	−0.08 (−0.26, 0.09)	0.361
- 36 Weeks	−0.12 (−0.36, 0.10)	0.341	−0.10 (−0.35, 0.15)	0.425
MTFM		0.239 ^†^		0.263 ^†^
- 28 Weeks	−0.10 (−0.33, 0.12)	0.370	−0.09 (−0.32, 0.14)	0.444
- 36 Weeks	−0.39 (−0.90, 0.12)	0.141	−0.37 (−0.90, 0.17)	0.177
AFM		0.377 ^†^		0.431 ^†^
- 28 Weeks	−0.07 (−0.23, 0.08)	0.357	−0.12 (−0.29, 0.04)	0.141
- 36 Weeks	−0.17 (−0.41, 0.08)	0.152	−0.21 (−0.44, 0.02)	0.075
SSFM		0.824 ^†^		0.930 ^†^
- 28 Weeks	−0.14 (−0.28, 0.00)	0.053	−0.17 (−0.32, −0.03)	0.021
- 36 Weeks	−0.17 (−0.39, 0.00)	0.141	−0.18 (−0.41, 0.04)	0.115

^†^ Denotes *p* value for test of interaction between HEI and time. That is, for a test of whether the association between HEI and fetal growth/adiposity at 28 weeks differs from the association at 36 weeks.

**Table 3:** Log Dietary Intake and fetal ultrasound measurements.

Outcome	Unadjusted Estimate (95% CI)	Unadjusted *p* Value	Adjusted Estimate (95% CI)	Adjusted *p* Value
BPD		0.116 ^†^		0.099 ^†^
- 28 Weeks	−0.31 (−1.48, 0.86)	0.603	−0.36 (−1.55, 0.82)	0.547
- 36 Weeks	−1.36 (−2.43, 0.86)	0.012	−1.48 (−2.55, −0.40)	0.007
BPD z score		0.417 ^†^		0.477 ^†^
- 28 Weeks	−0.34 (−4.39, 3.71)	0.869	−0.59 (−4.71, 3.54)	0.780
- 36 Weeks	−2.14 (−5.59, 3.71)	0.225	−2.18 (−5.70, 1.35)	0.226
HC		0.260 ^†^		0.169 ^†^
- 28 Weeks	−0.90 (−5.01, 3.22)	0.669	−0.72 (−4.84, 3.40)	0.732
- 36 Weeks	−3.64 (−7.17, 3.22)	0.043	−4.07 (−7.60, −0.54)	0.024
HC z score		0.390 ^†^		0.347 ^†^
- 28 Weeks	0.52 (−2.27, 3.31)	0.716	0.67 (−2.18, 3.52)	0.647
- 36 Weeks	−0.83 (−3.33, 3.31)	0.519	−0.83 (−3.36, 1.71)	0.524
FL		0.657 ^†^		0.570 ^†^
- 28 Weeks	−0.20 (−1.17, 0.76)	0.680	−0.22 (−1.20, 0.75)	0.653
- 36 Weeks	−0.47 (−1.40, 0.76)	0.327	−0.56 (−1.52, 0.39)	0.248
FL z score		0.785 ^†^		0.762 ^†^
- 28 Weeks	0.02 (−2.92, 2.97)	0.988	−0.06 (−3.07, 2.96)	0.970
- 36 Weeks	0.51 (−2.59, 2.97)	0.746	0.49 (−2.67, 3.65)	0.762
AC		0.246 ^†^		0.181 ^†^
- 28 Weeks	−0.21 (−5.45, 5.04)	0.938	0.81 (−4.23, 5.85)	0.753
- 36 Weeks	−3.83 (−9.40, 5.04)	0.178	−3.34 (−8.86, 2.19)	0.236
AC z score		0.860 ^†^		0.815 ^†^
- 28 Weeks	0.44 (−2.27, 3.16)	0.748	1.14 (−1.51, 3.78)	0.399
- 36 Weeks	0.18 (−2.73, 3.16)	0.905	0.78 (−2.16, 3.72)	0.603
EFW		0.082 ^†^		0.059 ^†^
- 28 Weeks	130.32 (−598.56, 859.21)	0.726	204.16 (−512.19, 920.51)	0.576
- 36 Weeks	−887.76 (−2026.25, 859.21)	0.126	−901.31 (−2028.21, 225.59)	0.117
EFW z score		0.305 ^†^		0.300 ^†^
- 28 Weeks	1.14 (−1.32, 3.59)	0.364	1.47 (−0.96, 3.91)	0.236
- 36 Weeks	−0.29 (−2.85, 3.59)	0.825	0.01 (−2.58, 2.61)	0.991
MTLM		0.495 ^†^		0.574 ^†^
- 28 Weeks	−4.56 (−9.20, 0.08)	0.054	−4.94 (−9.57, −0.32)	0.036
- 36 Weeks	−7.07 (−13.69, 0.08)	0.037	−7.02 (−13.69, −0.35)	0.039
MTFM		0.812 ^†^		0.795 ^†^
- 28 Weeks	−0.90 (−6.35, 4.55)	0.746	−1.76 (−7.35, 3.83)	0.538
- 36 Weeks	0.46 (−10.91, 4.55)	0.937	−0.25 (−11.82, 11.31)	0.966
AFM		0.563 ^†^		0.603 ^†^
- 28 Weeks	−1.00 (−5.03, 3.03)	0.627	−0.59 (−4.65, 3.48)	0.777
- 36 Weeks	0.88 (−5.59, 3.03)	0.791	1.10 (−5.27, 7.47)	0.734
SSFM		0.779 ^†^		0.760 ^†^
- 28 Weeks	2.72 (−0.73, 6.17)	0.122	3.23 (−0.22, 6.69)	0.067
- 36 Weeks	1.88 (−3.72, 6.17)	0.511	2.32 (−3.26, 7.90)	0.416

^†^ Denotes *p* value for interaction between time and log Total Energy; that is, for a test of whether the association between log Total Energy and fetal growth/adiposity at 28 weeks differs from the association at 36 weeks.

**Table 4:** Glycaemic Index and fetal ultrasound measurements.

Outcome	Unadjusted Estimate (95% CI)	Unadjusted *p* Value	Adjusted Estimate (95% CI)	Adjusted *p* Value
BPD		0.079 ^†^		0.060 ^†^
- 28 Weeks	−0.12 (−0.21, −0.02)	0.021	−0.11 (−0.21, −0.01)	0.035
- 36 Weeks	−0.01 (−0.11, −0.02)	0.876	0.01 (−0.09, 0.11)	0.885
BPD *z*-score		0.075 ^†^		0.083 ^†^
- 28 Weeks	−0.36 (−0.70, −0.02)	0.037	−0.35 (−0.69, −0.01)	0.045
- 36 Weeks	−0.03 (−0.31, −0.02)	0.812	−0.03 (−0.31, 0.25)	0.833
HC		0.601 ^†^		0.620 ^†^
- 28 Weeks	−0.19 (−0.54, 0.16)	0.288	−0.14 (−0.50, 0.21)	0.422
- 36 Weeks	−0.08 (−0.42, 0.16)	0.642	−0.04 (−0.38, 0.30)	0.816
HC *z*-score		0.652 ^†^		0.540 ^†^
- 28 Weeks	0.02 (−0.22, 0.26)	0.880	0.04 (−0.20, 0.29)	0.724
- 36 Weeks	−0.04 (−0.27, 0.26)	0.709	−0.04 (−0.26, 0.18)	0.723
FL		0.729 ^†^		0.634 ^†^
- 28 Weeks	−0.05 (−0.13, 0.03)	0.250	−0.05 (−0.13, 0.03)	0.236
- 36 Weeks	−0.03 (−0.12, 0.03)	0.521	−0.02 (−0.11, 0.07)	0.595
FL *z*-score		0.904 ^†^		0.931 ^†^
- 28 Weeks	−0.03 (−0.29, 0.23)	0.820	−0.04 (−0.30, 0.23)	0.790
- 36 Weeks	−0.01 (−0.31, 0.23)	0.949	−0.02 (−0.32, 0.27)	0.891
AC		0.185 ^†^		0.158 ^†^
- 28 Weeks	−0.24 (−0.66, 0.17)	0.248	−0.23 (−0.63, 0.17)	0.257
- 36 Weeks	0.10 (−0.34, 0.17)	0.649	0.13 (−0.30, 0.57)	0.556
AC *z*-score		0.151 ^†^		0.182 ^†^
- 28 Weeks	−0.09 (−0.31, 0.13)	0.422	−0.09 (−0.31, 0.12)	0.383
- 36 Weeks	0.09 (−0.12, 0.13)	0.396	0.07 (−0.14, 0.28)	0.491
EFW		0.583 ^†^		0.551 ^†^
- 28 Weeks	−18.94 (−79.38, 41.50)	0.539	−17.21 (−77.32, 42.90)	0.575
- 36 Weeks	8.76 (−89.14, 41.50)	0.861	12.58 (−84.02, 109.19)	0.799
EFW *z*-score		0.212 ^†^		0.247 ^†^
- 28 Weeks	−0.11 (−0.33, 0.10)	0.314	−0.11 (−0.32, 0.10)	0.316
- 36 Weeks	0.03 (−0.17, 0.10)	0.749	0.02 (−0.18, 0.22)	0.813
MTLM		0.706 ^†^		0.686 ^†^
- 28 Weeks	0.11 (−0.25, 0.46)	0.548	0.13 (−0.22, 0.49)	0.462
- 36 Weeks	−0.02 (−0.63, 0.46)	0.950	−0.00 (−0.61, 0.61)	0.993
MTFM		0.015 ^†^		0.025 ^†^
- 28 Weeks	−0.36 (−0.80, 0.07)	0.104	−0.34 (−0.77, 0.10)	0.133
- 36 Weeks	0.79 (−0.12, 0.07)	0.089	0.74 (−0.18, 1.65)	0.116
AFM		0.115 ^†^		0.150 ^†^
- 28 Weeks	−0.11 (−0.41, 0.19)	0.475	−0.13 (−0.44, 0.18)	0.415
- 36 Weeks	0.34 (−0.19, 0.19)	0.211	0.28 (−0.24, 0.81)	0.291
SSFM		0.215 ^†^		0.176 ^†^
- 28 Weeks	−0.06 (−0.35, 0.22)	0.661	−0.07 (−0.35, 0.22)	0.639
- 36 Weeks	0.25 (−0.21, 0.22)	0.287	0.28 (−0.19, 0.75)	0.248

^†^ Denotes *p* value for time-by-GI interaction; that is does the association between GI and fetal growth/adiposity at 28 weeks differ from that at 36 weeks.

**Table 5:** Glycaemic load and fetal ultrasound measurements.

Outcome	Unadjusted Estimate (95% CI)	Unadjusted *p* Value	Adjusted Estimate (95% CI)	Adjusted *p* Value
BPD		0.567 ^†^		0.490 ^†^
- 28 Weeks	−0.00 (−0.01, 0.00)	0.251	−0.00 (−0.01, 0.00)	0.276
- 36 Weeks	−0.01 (−0.01, 0.00)	0.063	−0.01 (−0.01, 0.00)	0.054
BPD *z*-score		0.821 ^†^		0.831 ^†^
- 28 Weeks	−0.01 (−0.04, 0.01)	0.227	−0.01 (−0.04, 0.01)	0.227
- 36 Weeks	−0.01 (−0.03, 0.01)	0.295	−0.01 (−0.03, 0.01)	0.291
HC		0.562 ^†^		0.374 ^†^
- 28 Weeks	−0.01 (−0.03, 0.02)	0.530	−0.01 (−0.03, 0.02)	0.683
- 36 Weeks	−0.02 (−0.04, 0.02)	0.137	−0.02 (−0.04, 0.00)	0.102
HC *z*-score		0.606 ^†^		0.479 ^†^
- 28 Weeks	0.00 (−0.02, 0.02)	0.964	0.00 (−0.02, 0.02)	0.808
- 36 Weeks	−0.01 (−0.02, 0.02)	0.557	−0.01 (−0.02, 0.01)	0.539
FL		0.827 ^†^		0.737 ^†^
- 28 Weeks	−0.00 (−0.01, 0.01)	0.698	−0.00 (−0.01, 0.01)	0.762
- 36 Weeks	−0.00 (−0.01, 0.01)	0.471	−0.00 (−0.01, 0.00)	0.437
FL *z*-score		0.676 ^†^		0.674 ^†^
- 28 Weeks	−0.00 (−0.02, 0.02)	0.923	−0.00 (−0.02, 0.02)	0.965
- 36 Weeks	0.00 (−0.02, 0.02)	0.688	0.00 (−0.01, 0.02)	0.653
AC		0.492 ^†^		0.391 ^†^
- 28 Weeks	−0.00 (−0.04, 0.03)	0.837	0.00 (−0.03, 0.04)	0.814
- 36 Weeks	−0.02 (−0.05, 0.03)	0.340	−0.01 (−0.05, 0.02)	0.465
AC *z*-score		0.861 ^†^		0.969 ^†^
- 28 Weeks	0.00 (−0.02, 0.02)	0.973	0.00 (−0.01, 0.02)	0.548
- 36 Weeks	0.00 (−0.02, 0.02)	0.835	0.01 (−0.01, 0.02)	0.604
EFW		0.181 ^†^		0.145 ^†^
- 28 Weeks	0.93 (−3.72, 5.59)	0.694	1.66 (−2.95, 6.27)	0.481
- 36 Weeks	−4.15 (−11.63, 5.59)	0.276	−3.95 (−11.48, 3.58)	0.304
EFW *z*-score		0.636 ^†^		0.567 ^†^
- 28 Weeks	0.00 (−0.01, 0.02)	0.717	0.01 (−0.01, 0.02)	0.459
- 36 Weeks	−0.00 (−0.02, 0.02)	0.857	0.00 (−0.02, 0.02)	0.980
MTLM		0.406 ^†^		0.462 ^†^
- 28 Weeks	−0.02 (−0.05, 0.00)	0.098	−0.02 (−0.05, 0.00)	0.093
- 36 Weeks	−0.04 (−0.08, 0.00)	0.052	−0.04 (−0.08, 0.00)	0.064
MTFM		0.252 ^†^		0.264 ^†^
- 28 Weeks	−0.02 (−0.05, 0.01)	0.262	−0.02 (−0.05, 0.01)	0.215
- 36 Weeks	0.02 (−0.05, 0.01)	0.522	0.02 (−0.05, 0.10)	0.578
AFM		0.278 ^†^		0.326 ^†^
- 28 Weeks	0.00 (−0.02, 0.03)	0.891	0.00 (−0.02, 0.03)	0.721
- 36 Weeks	0.02 (−0.02, 0.03)	0.244	0.03 (−0.02, 0.07)	0.223
SSFM		0.737 ^†^		0.757 ^†^
- 28 Weeks	0.01 (−0.01, 0.04)	0.185	0.02 (−0.00, 0.04)	0.106
- 36 Weeks	0.02 (−0.01, 0.04)	0.239	0.02 (−0.01, 0.06)	0.189

^†^ Denotes p value for interaction between time and Glycaemic Load; that is does the association between GL and fetal growth/adiposity at 28 weeks differ from that at 36 weeks.

**Table 6:** Fat as a percentage of total energy and fetal ultrasound measurements.

Outcome	Unadjusted Estimate (95% CI)	Unadjusted *p* Value	Adjusted Estimate (95% CI)	Adjusted *p* Value
BPD		0.593 ^†^		0.646 ^†^
- 28 Weeks	0.04 (−0.05, 0.12)	0.396	0.04 (−0.05, 0.12)	0.418
- 36 Weeks	0.01 (−0.08, 0.12)	0.841	0.01 (−0.08, 0.10)	0.793
BPD *z*-score		0.387 ^†^		0.507 ^†^
- 28 Weeks	0.21 (−0.08, 0.50)	0.152	0.17 (−0.12, 0.46)	0.238
- 36 Weeks	0.08 (−0.16, 0.50)	0.524	0.07 (−0.17, 0.31)	0.560
HC		0.083 ^†^		0.123 ^†^
- 28 Weeks	0.16 (−0.12, 0.44)	0.255	0.18 (−0.11, 0.46)	0.228
- 36 Weeks	−0.13 (−0.39, 0.44)	0.318	−0.09 (−0.35, 0.17)	0.499
HC *z*-score		0.016 ^†^		0.033 ^†^
- 28 Weeks	0.22 (0.01, 0.43)	0.036	0.21 (−0.00, 0.43)	0.053
- 36 Weeks	−0.05 (−0.23, 0.43)	0.570	−0.03 (−0.21, 0.14)	0.714
FL		0.413 ^†^		0.560 ^†^
- 28 Weeks	0.00 (−0.07, 0.07)	0.896	0.00 (−0.07, 0.07)	0.950
- 36 Weeks	−0.03 (−0.10, 0.07)	0.381	−0.02 (−0.09, 0.04)	0.514
FL *z*-score		0.414 ^†^		0.577 ^†^
- 28 Weeks	0.06 (−0.15, 0.28)	0.563	0.03 (−0.19, 0.24)	0.808
- 36 Weeks	−0.04 (−0.26, 0.28)	0.683	−0.05 (−0.26, 0.17)	0.664
AC		0.556 ^†^		0.609 ^†^
- 28 Weeks	−0.02 (−0.37, 0.33)	0.920	0.03 (−0.32, 0.39)	0.853
- 36 Weeks	−0.15 (−0.53, 0.33)	0.428	−0.08 (−0.46, 0.29)	0.660
AC *z*-score		0.968 ^†^		0.806 ^†^
- 28 Weeks	−0.02 (−0.21, 0.16)	0.799	−0.01 (−0.20, 0.18)	0.922
- 36 Weeks	−0.02 (−0.21, 0.16)	0.833	0.02 (−0.17, 0.20)	0.851
EFW		0.253 ^†^		0.307 ^†^
- 28 Weeks	14.86 (−35.95, 65.68)	0.566	18.79 (−33.86, 71.44)	0.484
- 36 Weeks	−34.32 (−113.89, 65.68)	0.398	−25.56 (−104.88, 53.76)	0.528
EFW *z*-score		0.308 ^†^		0.477 ^†^
- 28 Weeks	0.05 (−0.12, 0.22)	0.567	0.05 (−0.13, 0.22)	0.610
- 36 Weeks	−0.05 (−0.21, 0.22)	0.551	−0.02 (−0.19, 0.14)	0.771
MTLM		0.446 ^†^		0.372 ^†^
- 28 Weeks	0.07 (−0.26, 0.40)	0.669	0.09 (−0.25, 0.44)	0.602
- 36 Weeks	−0.15 (−0.67, 0.40)	0.565	−0.18 (−0.70, 0.35)	0.511
MTFM		0.287 ^†^		0.284 ^†^
- 28 Weeks	0.03 (−0.38, 0.44)	0.882	0.04 (−0.38, 0.47)	0.837
- 36 Weeks	−0.40 (−1.12, 0.44)	0.281	−0.39 (−1.12, 0.33)	0.290
AFM		0.049 ^†^		0.060 ^†^
- 28 Weeks	0.01 (−0.26, 0.29)	0.917	0.06 (−0.24, 0.36)	0.709
- 36 Weeks	−0.46 (−0.90, 0.29)	0.041	−0.39 (−0.82, 0.04)	0.075
SSFM		0.368 ^†^		0.295 ^†^
- 28 Weeks	0.15 (−0.08, 0.37)	0.200	0.17 (−0.06, 0.40)	0.144
- 36 Weeks	−0.03 (−0.39, 0.37)	0.863	−0.04 (−0.40, 0.32)	0.829

^†^ denotes *p* value for test of interaction between fat % and time. That is, whether the association at 28 weeks differs from that at 36 weeks.

**Table 7:** Carbohydrate as a percentage of total energy and fetal ultrasound measurements.

Outcome	Unadjusted Estimate (95% CI)	Unadjusted *p* Value	Adjusted Estimate (95% CI)	Adjusted *p* Value
BPD		0.339 ^†^		0.381 ^†^
- 28 Weeks	−0.03 (−0.10, 0.05)	0.482	−0.02 (−0.09, 0.06)	0.634
- 36 Weeks	0.02 (−0.05, 0.05)	0.653	0.02 (−0.05, 0.09)	0.554
BPD *z*-score		0.156 ^†^		0.241 ^†^
- 28 Weeks	−0.16 (−0.38, 0.05)	0.143	−0.13 (−0.35, 0.09)	0.262
- 36 Weeks	0.01 (−0.17, 0.05)	0.883	0.02 (−0.16, 0.21)	0.819
HC		0.199 ^†^		0.306 ^†^
- 28 Weeks	−0.08 (−0.31, 0.15)	0.499	−0.05 (−0.28, 0.19)	0.685
- 36 Weeks	0.10 (−0.11, 0.15)	0.354	0.09 (−0.11, 0.29)	0.374
HC *z*-score		0.099 ^†^		0.188 ^†^
- 28 Weeks	−0.10 (−0.26, 0.05)	0.200	−0.08 (−0.24, 0.08)	0.331
- 36 Weeks	0.05 (−0.09, 0.05)	0.503	0.04 (−0.10, 0.18)	0.562
FL		0.849 ^†^		0.816 ^†^
- 28 Weeks	0.02 (−0.04, 0.08)	0.480	0.03 (−0.03, 0.08)	0.336
- 36 Weeks	0.01 (−0.03, 0.08)	0.579	0.02 (−0.03, 0.07)	0.415
FL *z*-score		0.996 ^†^		0.943 ^†^
− 28 Weeks	0.04 (−0.13, 0.20)	0.652	0.07 (−0.10, 0.23)	0.432
- 36 Weeks	0.04 (−0.12, 0.20)	0.651	0.06 (−0.10, 0.22)	0.470
AC		0.913 ^†^		0.782 ^†^
- 28 Weeks	0.05 (−0.26, 0.35)	0.771	0.10 (−0.21, 0.41)	0.532
- 36 Weeks	0.03 (−0.25, 0.35)	0.853	0.05 (−0.23, 0.33)	0.729
AC *z*-score		0.751 ^†^		0.482 ^†^
- 28 Weeks	0.03 (−0.12, 0.18)	0.732	0.06 (−0.09, 0.21)	0.420
- 36 Weeks	−0.00 (−0.14, 0.18)	0.983	−0.00 (−0.14, 0.14)	0.994
EFW		0.962 ^†^		0.976 ^†^
- 28 Weeks	7.29 (−36.60, 51.19)	0.745	16.48 (−28.72, 61.67)	0.475
- 36 Weeks	8.87 (−50.10, 51.19)	0.768	15.46 (−43.22, 74.14)	0.606
EFW *z*-score		0.777 ^†^		0.953 ^†^
- 28 Weeks	0.00 (−0.13, 0.14)	0.979	0.04 (−0.10, 0.17)	0.611
- 36 Weeks	0.03 (−0.10, 0.14)	0.699	0.03 (−0.10, 0.16)	0.643
MTLM		0.867 ^†^		0.838 ^†^
- 28 Weeks	−0.06 (−0.33, 0.20)	0.639	−0.04 (−0.31, 0.23)	0.783
- 36 Weeks	−0.02 (−0.40, 0.20)	0.901	0.01 (−0.37, 0.39)	0.961
MTFM		0.406 ^†^		0.406 ^†^
- 28 Weeks	−0.10 (−0.44, 0.24)	0.558	−0.07 (−0.41, 0.27)	0.683
- 36 Weeks	0.17 (−0.41, 0.24)	0.563	0.20 (−0.38, 0.79)	0.495
AFM		0.118 ^†^		0.173 ^†^
- 28 Weeks	0.04 (−0.18, 0.26)	0.732	0.06 (−0.17, 0.28)	0.614
- 36 Weeks	0.32 (−0.00, 0.26)	0.051	0.30 (−0.02, 0.62)	0.062
SSFM		0.800 ^†^		0.836 ^†^
- 28 Weeks	0.00 (−0.18, 0.18)	0.966	0.01 (−0.17, 0.19)	0.879
- 36 Weeks	0.04 (−0.23, 0.18)	0.755	0.05 (−0.23, 0.32)	0.738

^†^ Denotes *p* value for test of interaction between Carbohydrate % and fetal growth/adiposity; that is, whether the association at 28 weeks differs from that at 36 weeks.

**Table 8:** Protein as a percentage of total energy and fetal ultrasound measurements.

Outcome	Unadjusted Estimate (95% CI)	Unadjusted *p* Value	Adjusted Estimate (95% CI)	Adjusted *p* Value
BPD		0.507 ^†^		0.546 ^†^
- 28 Weeks	0.01 (−0.10, 0.11)	0.921	−0.01 (−0.11, 0.10)	0.914
- 36 Weeks	−0.03 (−0.12, 0.11)	0.466	−0.04 (−0.13, 0.05)	0.361
BPD *z*-score		0.153 ^†^		0.210 ^†^
- 28 Weeks	0.13 (−0.18, 0.44)	0.414	0.10 (−0.21, 0.42)	0.522
- 36 Weeks	−0.10 (−0.35, 0.44)	0.400	−0.11 (−0.35, 0.14)	0.399
HC		0.991 ^†^		0.802 ^†^
- 28 Weeks	−0.05 (−0.38, 0.27)	0.755	−0.12 (−0.45, 0.22)	0.489
- 36 Weeks	−0.05 (−0.33, 0.27)	0.723	−0.07 (−0.34, 0.20)	0.618
HC *z*-score		0.983 ^†^		0.806 ^†^
- 28 Weeks	−0.05 (−0.26, 0.16)	0.621	−0.08 (−0.30, 0.13)	0.440
- 36 Weeks	−0.05 (−0.23, 0.16)	0.588	−0.05 (−0.24, 0.13)	0.567
FL		0.212 ^†^		0.269 ^†^
- 28 Weeks	−0.06 (−0.14, 0.02)	0.143	−0.06 (−0.14, 0.01)	0.110
- 36 Weeks	0.00 (−0.07, 0.02)	0.994	−0.01 (−0.08, 0.05)	0.692
FL *z*-score		0.499 ^†^		0.633 ^†^
- 28 Weeks	−0.14 (−0.37, 0.09)	0.224	−0.14 (−0.36, 0.09)	0.235
- 36 Weeks	−0.04 (−0.26, 0.09)	0.719	−0.07 (−0.29, 0.15)	0.547
AC		0.264 ^†^		0.187 ^†^
- 28 Weeks	−0.16 (−0.60, 0.29)	0.490	−0.29 (−0.74, 0.16)	0.208
- 36 Weeks	0.13 (−0.28, 0.29)	0.531	0.06 (−0.35, 0.47)	0.785
AC *z*-score		0.501 ^†^		0.281 ^†^
- 28 Weeks	−0.06 (−0.27, 0.15)	0.584	−0.13 (−0.34, 0.08)	0.211
- 36 Weeks	0.02 (−0.18, 0.15)	0.839	−0.00 (−0.21, 0.21)	0.992
EFW		0.175 ^†^		0.173 ^†^
- 28 Weeks	−40.93 (−105.51, 23.64)	0.214	−56.34 (−123.01, 10.33)	0.098
- 36 Weeks	22.54 (−62.18, 23.64)	0.602	7.75 (−76.06, 91.57)	0.856
EFW *z*-score		0.478 ^†^		0.351 ^†^
- 28 Weeks	−0.09 (−0.28, 0.10)	0.350	−0.14 (−0.33, 0.05)	0.155
- 36 Weeks	−0.01 (−0.20, 0.10)	0.915	−0.03 (−0.22, 0.16)	0.753
MTLM		0.433 ^†^		0.395 ^†^
- 28 Weeks	0.09 (−0.26, 0.44)	0.617	0.05 (−0.31, 0.41)	0.800
- 36 Weeks	0.33 (−0.18, 0.44)	0.201	0.31 (−0.19, 0.81)	0.227
MTFM		0.833 ^†^		0.823 ^†^
- 28 Weeks	0.08 (−0.35, 0.51)	0.711	0.07 (−0.37, 0.51)	0.765
- 36 Weeks	−0.02 (−0.91, 0.51)	0.968	−0.04 (−0.93, 0.85)	0.932
AFM		0.467 ^†^		0.661 ^†^
- 28 Weeks	−0.10 (−0.41, 0.22)	0.548	−0.17 (−0.49, 0.14)	0.288
- 36 Weeks	−0.26 (−0.68, 0.22)	0.216	−0.27 (−0.68, 0.14)	0.194
SSFM		0.872 ^†^		0.736 ^†^
- 28 Weeks	−0.18 (−0.44, 0.09)	0.189	−0.21 (−0.48, 0.05)	0.119
- 36 Weeks	−0.14 (−0.57, 0.09)	0.524	−0.13 (−0.55, 0.29)	0.550

^†^ Denotes p value for test of interaction between time and Protein %; that is, whether the association at 28 weeks differs from that at 36 weeks.
